# Modernizing Relationship Therapy through Social Thermoregulation Theory: Evidence, Hypotheses, and Explorations

**DOI:** 10.3389/fpsyg.2017.00635

**Published:** 2017-05-01

**Authors:** Hans IJzerman, Emma C. E. Heine, Saskia K. Nagel, Tila M. Pronk

**Affiliations:** ^1^Department of Psychology, University of Virginia, CharlottesvilleVA, USA; ^2^Department of Clinical Psychology, Vrije Universiteit AmsterdamAmsterdam, Netherlands; ^3^Department of Philosophy, University of TwenteEnschede, Netherlands; ^4^Department of Social and Organisational Psychology, Tilburg UniversityTilburg, Netherlands

**Keywords:** social thermoregulation, attachment, relationship therapy, emotion regulation, wearables, sensor technology, actuators

## Abstract

In the present article the authors propose to modernize relationship therapy by integrating novel sensor and actuator technologies that can help optimize people’s thermoregulation, especially as they pertain to social contexts. Specifically, they propose to integrate *Social Thermoregulation Theory* (IJzerman et al., 2015a; IJzerman and Hogerzeil, 2017) into Emotionally Focused Therapy by first doing exploratory research during couples’ therapy, followed by Randomized Clinical Trials (RCTs). The authors thus suggest crafting a *Social Thermoregulation Therapy* (STT) as enhancement to existing relationship therapies. The authors outline what is known and not known in terms of social thermoregulatory mechanisms, what kind of data collection and analyses are necessary to better understand social thermoregulatory mechanisms to craft interventions, and stress the need to conduct RCTs prior to implementation. They further warn against too hastily applying these theoretical perspectives. The article concludes by outlining why STT is the way forward in improving relationship functioning.

## Introduction

One of the strongest predictors of one’s physical health, mental health, and happiness is the quality of one’s close relationships. Having high quality relationships predicts factors that we understand as *life chances*, including a longer life, greater creativity, and higher self-esteem (House et al., 1988; Argyle, 1992; Holt-Lunstad et al., 2010). However, to date, our understanding of why high quality social relationships lead to a more fulfilled and healthy life is relatively limited. The present paper serves to provide further direction to understanding some prominent underlying mechanisms through *social thermoregulation theory.* In addition, we will outline how near-future interventions can be crafted by doing research with novel technologies during relationship therapy.

Thus far, the evidence linking relationships and life chances focused at “higher order” levels: marital couples that regulate each other’s emotions successfully have fewer marital problems, have better health, and are more satisfied with their relationship than couples who do not successfully co-regulate (Gottman and Levenson, 1992). But our position is broader: first, disturbances in health closely relate to dysregulated body temperature (Benzinger, 1969). Second, temperature regulation has been a major driving force for sociality in homeothermic (= warm-blooded) animals (Ebensperger, 2001). For humans, the aggregate evidence is similarly in favor of an evolved reliance of social warmth on physical warmth (IJzerman et al., 2015a). Finally, the literature is in favor of the idea of *co-regulation*, a lower level dynamic that can help down-regulate emotional states socially (Butler and Randall, 2013).

The present article brings together these three concepts and asks the question *if* thermoregulation is crucial for physiological co-regulation in close relationships, and, consequently, proceeds to ask *whether* therapists can help improve physiological co-regulation in couples. Altogether, we propose to rely on novel technologies that can aid in developing *Social Thermoregulation Therapy* (STT) to help optimize people’s social lives.

In this article, we first provide what we see as one of the main functions of relationships: relationships help distribute the burdens of the environment and help to *co-regulate.* Then, we provide the available evidence on social thermoregulation theory, integrate co-regulation with social thermoregulation theory, after which we discuss potential interventions to improve *co-thermoregulation.* Most prominently, we point to modern sensor and actuator technology as tools to help develop and then implement STT. We clarify what we know and don’t know, followed by some of the risks we perceive in moving forward with such novel therapies. We anticipate that this approach will dramatically reduce the gap between researchers (theory) and therapists (application). Our position paper is much needed, as advances in this field will likely be so rapid that consequential mistakes in crafting novel relationship therapies are not unimaginable and potentially disastrous.

## The General Premise: Relationships are for Co-Regulation

In a seminal 1992 article, Gottman and Levenson (1992) found that *co-regulation* is crucial for a relationship’s success. They found that positive exchanges (e.g., responses through humor or positive problem descriptions rather than a negative, defensive response) toward a marriage partner were predictive of lower chance on divorce later, better health, and greater finger amplitude (indicative of autonomic activation). In the early days of this research, co-regulation was mostly understood through the regulation of emotions at higher, more conscious forms of attending to the other’s emotion (e.g., through humor or positive problem descriptions). With more advanced equipment, researchers have also started to pay greater attention to lower level dynamics that used to be much harder to capture. As but one example, Coan et al. (2006) found that simply holding the partner’s hand while under distress decreased stress-related activation in the brain while under threat of electric shock.

These insights on lower level dynamics led Butler and Randall (2013) to redefine co-regulation as the “bidirectional linkage of oscillating emotional channels (subjective experience, expressive behavior, and autonomic physiology) between partners (a linkage that) contributes to emotional and physiological stability for both partners in a close relationship” (p. 203), which thus incorporates lower level (autonomic) regulation with more conscious forms. Butler and Randall’s (2013) perspective supplements the early views imparted by Gottman and Levenson (1992) with a type of social emotion regulation that is less “in the head” and more distributed and dynamic, relying on an “affective attunement” between close partners (e.g., romantic partners or caregiver and infant).

The general aim of such affective attunement is to achieve an allostatic balance in the relationship through distributing risks of environmental threats, leading to an offloading of energetic demands created by such threats (e.g., Beckes and Coan, 2011; Fitzsimons et al., 2015). The field of behavioral ecology has illustrated this idea of load sharing with conspecifics. Ostriches, for example, increase the rate of eating when they are in the presence of other ostriches, which can look out for predators (Bertram, 1980; Krebs and Davies, 1993). Homeothermic animals, like rodents, huddle up to other animals when cold to offload the energetic demands of warming up (Ebensperger, 2001). Thus, beyond distributing threat, one of the constant and very demanding threats to allostatic balance is the near-constant change in environmental temperature. For most animals their ilk help downregulate the environmental challenge that fluctuations of temperature pose on them.

## *Why* Social Thermoregulation is Vital for Co-Regulation: The Available Evidence

Despite modern conveniences like heaters or cloths, temperature regulation remains a considerable challenge for humans. From that perspective, Social Thermoregulation Theory complements basic approaches to co-regulation, detailing how “social warmth” (i.e., trustworthiness and social predictability) relies on more ancient needs of physical warmth. Strong evidence for the relationship between social interaction and thermoregulation can be found in studies across homoeothermic animals. In rodents, social thermoregulation has been shown to be one of the most important motivating forces behind group living, especially when temperatures drop (Ebensperger, 2001). As but one example, the Octodon Degus (a Chilean rodent) used 40% less energy and achieved a higher surface temperature when housed with three or five others (versus alone; Nuñez-Villegas et al., 2014). Studies of vervet monkeys show somewhat more complex mechanisms, with larger social networks buffering their core temperatures from the cold (McFarland et al., 2015), while even grooming a dead vervet monkey’s pelt insulates against temperature variations (McFarland et al., 2015).

For humans, the aggregate evidence is similarly in favor of the evolved reliance of social on physical warmth. Psychological research has consistently shown that temperature fluctuations (either outside or lab temperature) is causally tied to social behaviors ranging from renting romance movies (Hong and Sun, 2012) to house-purchasing decisions (Van Acker et al., 2016) to basic effects on perception, language use, and memory (IJzerman and Semin, 2009; Schilder et al., 2014; Messer et al., 2017). The effect also works the other way around: if people feel the environment to be socially unpredictable, they perceive temperatures as lower, whereas the reverse is true if people feel psychologically safe (Zhong and Leonardelli, 2008; IJzerman and Semin, 2010; IJzerman et al., 2015b, 2016; Ebersole et al., 2016). The link between psychological safety and thermoregulation extends to consumer behavior: brands that are regarded as more trustworthy induce perceptions of higher temperature, while the degree to which one is affected by temperature determines what one would pay for the brand (IJzerman et al., 2015b). This led IJzerman et al. (2015b) to conclude that temperature perceptions are a sort of social “weather report,” or a temperature prediction system on the basis of which people know whether to rely on their social context (or not)^1^.

Although it *seems* unlikely that social thermoregulation is still heavily involved in adult social interactions, one has to note that the evolutionary window of availability of modern conveniences (like heaters and clothes) to regulate temperature has likely been too brief to make a noticeable difference in the reliance of social on physical warmth. As a result, the need for physical warmth likely has formed as a model, or template, through which humans come to understand and interpret their social interactions.

Accordingly, interaction with others outside people’s direct relationships should similarly rely on “temperature estimates.” And indeed, in humans (unlike penguins) social thermoregulation is not just about huddling, but instead about attaching to different people in different contexts. Perhaps the most compelling evidence on attaching in a variety of contexts from recent work on social integration and climatic variation. IJzerman et al. (2017b) found in a relatively large sample in 12 different countries that the lower people’s core temperatures, the more they engage in *complex social integration* (i.e., engage in contact with different people in different social contexts); they also found that this integration buffers their core against distance from the equator (as a proxy for colder climates). In short, the available evidence is strongly in favor of the idea that people’s social networks – even the more complex ones – protect them from the cold, and that humans adapt their social behaviors and cognitions to temperature changes.^2^

## *How* Social Thermoregulation Supports Co-Regulation: Evidence and Speculations

We have reviewed evidence that temperature affects our social behavior and cognitions in myriad ways, while we have also reviewed evidence that shows that complex social networks still protect us against the cold. But at present, it is still unclear exactly *how* humans help regulate each other’s temperature through more complex dynamics, if at all. Although there is now considerable evidence that social thermoregulation is (causally) tied to social cognitions and behaviors, the literature regarding co-thermoregulatory patterns is scarce. At best, we can extract some elementary effects and speculate about further mechanisms. Despite the limited evidence, we feel comfortable providing some first direction given the current state of diverse, but converging literatures.

For example, emotions like anxiety and sadness have come to be associated with lowered peripheral temperature (Ziegler and Cash, 1938; Ekman et al., 1983; McFarland, 1985; Ekman, 1993; Nummenmaa et al., 2014). Relatedly, adults’ peripheral temperatures drop when they feel socially excluded (IJzerman et al., 2012).^3^ Peripheral temperature changes also extend to early social interactions: when a mother leaves the room in the Strange Situation, the infant’s skin temperature drops. Skin temperature only returns to baseline once the mother returns (and not so when a stranger enters the room; Mizukami et al., 1990). Further, people respond to close others’ sadness (either partners or infants) with an increase in peripheral temperature (Vuorenkoski et al., 1969; IJzerman et al., 2015a). That these effects may be co-regulatory in nature could be inferred from studies that show that physical warmth downregulate the need for social contact after a lack of social warmth (IJzerman et al., 2012; Zhang and Risen, 2014).^4,5^

Why is the regulation of body temperature so important to our social regulation systems? Human infants – like many other altricial species – are not able to regulate their temperature independently and need to rely on the caregiver to thermoregulate. Early attachment processes of the human child are thus focused on its need to keep warm, likely forming the basis for an evolved model, or, rather, template, for mental (attachment-like) models concerning the relationship between physical and social warmth. Experimental evidence supports the temperature-template-attachment view: attachment has been found to moderate people’s responses to temperature cues in a variety of reports (see, e.g., IJzerman et al., 2013). Furthermore, Vergara et al. (2017, unpublished) found that individual differences in need for social thermoregulation and preference for temperature predict not only individual differences in attachment but also stress and health, providing further support for thermoregulation as essential feature of our attachment system.^6^

Thermoregulation – across animals – is crucial for survival. The available evidence in humans also points to a robust link with social behavior and cognition, one that seems to be crucial for attachment. We therefore strongly suspect that thermoregulation becomes integrated into higher-order prediction systems and that this “temperature prediction system” supports us in navigating our social environment. Trustworthiness of brands for example do not only increase temperature perceptions, they also drive purchasing decisions (IJzerman et al., 2015b), while temperature fluctuations also influence people’s conformity to the majority appeal (Huang et al., 2014) or their decisions to engage in social interactions (Hong and Sun, 2012; Van Acker et al., 2016).

And there are some indications that responses to others’ emotions manifest through peripheral temperature changes. This is why, in line with previous work (IJzerman et al., 2015b), we have reason to believe that the “weather report” we have used as a metaphor relies on peripheral temperature to provide people with information on the basis of which they adapt to social situations. “Spending” this on others should thus only happen if we expect to be “paid back” in the future. Wagemans and IJzerman (2015, unpublished) for example found that peripheral temperature increases, but *only* if the relationship is communal. Szymkow et al. (2013) and IJzerman et al. (2015b) find that people estimate temperature higher, but *only* if the target is trustworthy (and *only* if lab temperatures are lower; Ebersole et al., 2016; IJzerman et al., 2016). Finally, people’s need to thermoregulate is higher, but again *only* if they perceive others as trustworthy (i.e., are securely attached; Vergara et al., 2017, unpublished).

In other words, there is considerable variation in the degree to which we (literally) warm up to others. There is also variation in the degree to which we perceive benefits from others in relation to thermoregulation and consequently the degree to which people “spend” their thermoregulation on others. This “spending” should be contingent not only on one’s past experiences, but also on the quality of the relationship. With novel technological inventions it becomes possible to study these dynamics in a methodologically sound fashion, cost efficiently, and in *real time*.

## Social Thermoregulation’s Physiological Mechanisms: Toward Predicting Dynamic Patterns

The key to understanding temperature prediction systems – and how they help us adapt to social contexts – is the *economy of action* (Proffitt, 2006; Schnall et al., 2010; Beckes and Coan, 2011; Coan and Sbarra, 2015). The premise is simple: organisms need to take in more energy than they exert, and overspending the energy expenditure budget is a threat to allostatic balance. In other animals, the metabolic costs of thermoregulation are decreased when regulated socially (Gilbert et al., 2006). We believe that social emotion regulation is (partly) rooted in the need to maintain temperature homeostasis and that helping to regulate another’s sadness will *cost* to support if our own periphery rises in peripheral temperature. We will thus *only* offer emotion regulation if we suspect the other to “pay back” in the future (and we ask, is the relationship with the other is communal?).

In other words, the ‘economy’ of relationships can be understood by calculating who in the social network “pays” for survival and – in more modern days and relationships – who “pays” by facilitating day-to-day emotional functioning. Human relationships are therefore a bit like being modern-type penguins, but then in the sense that people’s “modern” emotional systems are reliant on much older (penguin) systems. We think that this modern emotional system could rely on a “temperature monetizing system” that helps us regulate and bargain toward temperature homeostasis (Satinoff, 1978, 1982; Anderson, 2010). At present, there is virtually no research detailing how thermoregulation and metabolism relate to social emotion regulation, but there is some support for the fact that attachment-like processes rely on metabolic regulation. For example, people who are more avoidant in their relationship orientation have higher levels of fasting glucose, indicating a higher reliance on their own metabolic resources (Coan and Sbarra, 2015; Ein-Dor et al., 2015; see also Henriksen et al., 2014; IJzerman et al., 2015a).

### Relationships and Co-thermoregulation

One of the goals of a relationship is thus to maintain a form of “temperature homeostasis”; keeping track of the health of the relationship through temperature helps us maintain an optimized social network. Despite the considerable evidence linking thermoregulation to social behaviors and cognitions, the underlying dynamics we need to understand to effectively integrate social thermoregulation theory into therapy are still highly speculative. We know that our need for social warmth relies on our need for physical warmth; we also know that the lack of high quality relationships is metabolically costly; we further know that high quality relationships protect us from the cold; and we also know that both experiencing emotions ourselves and seeing emotions in others are associated with peripheral temperature changes in ourselves. Based on these different, but converging literatures, we thus strongly suspect that people co-thermoregulate close others by warming one’s skin or hugging the other when sad, and that both acts are metabolically costly. Yet, whether this is true, and how they exactly interrelate is not at all clear.^7^

We further strongly suspect that co-thermoregulation can be responsive or unresponsive, based on how reliable the partners perceive the relationship to be (communality), or how reliable they themselves perceive the world in general to be (attachment style). With “responsive co-thermoregulation” – a new term we would like to introduce for relationship research and therapy – we mean the (non-conscious) regulation of each other’s temperature toward homeostasis in dyads. The degree to which one participates is thus contingent upon *perceived social predictability* (i.e., a combination between attachment and communality of the relationship). Unresponsive co-thermoregulation would thus imply *not* hugging the partner when he or she is sad, and *not* increasing peripheral temperature when the other is in need. What constitutes responsive and unresponsive co-thermoregulation is still in need of very specific classification.

Acknowledging that relationships are complex and that multiple factors contribute to successful regulation, further caution is warranted in applying this perspective on co-thermoregulation in therapy. That is, the perceived social predictability can be accurate or inaccurate as in some situations being non-responsive to one’s partner’s emotions might be *functional.* When one’s partner has a very bad temper or can be abusive, avoiding one’s partner’s anger – as opposed to engaging – can be considered more beneficial. Thus, part of classifying responsive versus unresponsive co-thermoregulation is the understanding of the social context in which co-thermoregulation occurs.

## From Speculation to Application: The Role of Technological Advancements

We have acknowledged that the dynamics of co-thermoregulation are yet unclear. Specifically, it is unclear how strong, in which situation, and for which types of emotion one’s peripheral temperature should in- or decrease. At the same time, the available evidence supports the idea that understanding co-thermoregulation is vital to achieve optimal social functioning. Thermoregulation has further been implicated in (mental) health, such as depression (Pechlivanova et al., 2010), insomnia (Bach et al., 2002; Van den Heuvel et al., 2004; Pechlivanova et al., 2010), anxiety (Parry, 2007), and many others. Furthermore, physiological processes related to thermoregulation (like catabolization of *Brown Adipose Tissue*) have become linked to tumor growth (Shellock et al., 1986; Lee et al., 2010) or late onset obesity (Himms-Hagen, 1979, 1989, 1990; Van Marken Lichtenbelt et al., 2009). In other words, proper (social) thermoregulation seems vital for having optimal health.

Relationships, health, and thermoregulation are strongly interdependent, and understanding and applying thermoregulation may well-present one of the most important advances in modern (relationship) therapy. The reason why integrating thermoregulation into modern therapy has become feasible is because of advances in a field called “eHealth” (short for electronic health), a field that has become trendy in clinical research, mostly due to obvious benefits in saving costs, time, and the lower threshold to receive therapy. The most common applications of eHealth have been to seek a reduction of costs, by for example moving part of the therapy process online (and thus decreasing the amount of hours invested in providing therapy or assessments). For STT, eHealth can also quickly help us decrease costs of research by advancing our understanding through measurements. Could it be that – because of all the intimate links between relationships, thermoregulation, and health, that STT can quickly and fundamentally transform and optimize the type of care we can receive, thereby optimize the quality of our social networks?

### Application of Co-thermoregulation into Current Therapies

The application of STT into eHealth is likely most efficacious by adding it to an existing intervention known as *Emotionally Focused Therapy* (EFT). EFT is a short-term relationship therapy focusing on (co-regulatory) patterns in interaction (Johnson, 1999; Johnson et al., 1999; Greenberg, 2004). Various potential patterns of interaction in relationships are described and targeted through this type of therapy, one that is rooted in attachment theory (Bowlby, 1969). One example that shows these dynamics and its roots in attachment theory is the pattern that details how quality and emotionally unresponsive interactions often leading to stonewalling or emotionally “attacking” each other in the relationship (like Gottman and Levenson’s, 1992, classification a *non-regulated couple*).

Some have claimed EFT to be the most researched and most effective couple’s therapy (Johnson et al., 1999; Wiebe and Johnson, 2016), with 10 sessions of EFT improving dyadic adjustment of the relationship, and others regard it as a form of exposure therapy, exposing the couples to experiences that are emotionally taxing within the relationship (Greenman and Johnson, 2013). A few sessions of EFT have also been found to elicit greater emotional dependency on the partner (allowing to “distribute” the risk), as handholding after EFT reduced the stress experienced after electric shock through Coan et al.’s (2006) handholding paradigm (Johnson et al., 2013). Johnson et al.’s (1999) ideas are reminiscent of Gottman and Levenson’s (1992) idea of the “regulated couple” where a positive marital exchange, as a “bidirectional linkage of oscillating patterns…(between partners)” contributes to the marriage’s success. In more recent research, this view was supplemented with lower level interactions, not only by being vulnerable and offloading stress to others (Beckes and Coan, 2011; Butler and Randall, 2013), but now also by our proposal to offloading temperature regulation to the environment through what we have called the “temperature monetizing system.”

At present, we know that people in high quality relationships increase in peripheral temperature when the other is stressed (Vuorenkoski et al., 1969). The central proposal of STT would be to *adjust* (i.e., re-associate) peripheral temperatures in a relationship to specific social situations but *only* if one’s perception of social predictability within the relationship is misaligned. One could thus liken STT to better known neurofeedback paradigms (e.g., Lubar et al., 1995). Altering one’s peripheral temperature without attention to context will certainly not reliably alter the relationship dynamic. Integrating STT into EFT in other words is complex. Not only is it hard for therapists to assess the level of co-regulation in real life, at present it is still unclear when temperature in- or decreases (and how strongly) occur in communal relationships to different emotions by the partner, and it is thus unclear *when* co-thermoregulation is responsive and when not. Furthermore, *some* types of emotions are probably reliant on co-regulation (e.g., a “cooling” state like sadness) whereas this may not be true for other emotions (e.g., a “heating” state like anger).^8^ In addition, it is unclear how frequently one should manipulate peripheral temperature to support the relationship more permanently. What is clear is that STT has the potential to transform EFT by seamlessly tracking couples’ physiology in daily life.

Finally, STT is *not* a replacement for therapy related to higher order cognition, but should complement existing therapies (like Cognitive Behavioral Therapy and EFT) by addressing lower level dynamics. This is also why not all couples may benefit from aiding the relationship for the sake of staying together. Some clients might be at the end of a relationship and be better off having the therapist mediate their separation than putting time and effort in trying to save the relationship. The challenges seem various and daunting. But we suppose most of these issues are resolvable. We will now outline the steps to create the most efficacious STT by doing research during therapists’ EFT sessions with couples.

## Getting From Here to there: Research Therapists Can Do

With the advent of novel technologies, the gap between research and therapy decreases dramatically. For that reason, we describe how thermoregulatory dynamics can be researched during EFT sessions. We hope that interventions can be created based on this research. Between therapy sessions, therapists and researchers can monitor couples’ temperature, location, and proximity continuously for longer periods of time from a distance while the couples live their regular lives. Whereas most eHealth focuses on becoming more efficient in therapy, once the mechanisms are clearly defined, such *real time* monitoring can have considerable (practical) transformative consequences as compared to normal EFT, because therapists can start tapping into lower level dynamics. Again, exactly how this could be achieved needs to be researched in between therapy sessions. One of the advantages is that once protocols for STT are developed, the therapist will not simply have to recreate difficult and emotional interactions, but can instead track his or her clients in their daily lives.

The tools to start such a research endeavor between EFT sessions with tracking are within reach: smartphones and smartwatches have become available with accurate temperature sensors that can measure continuously and store data online or on a distant server (Park and Chen, 2007; Dufau et al., 2011; Aram et al., 2012; Lee et al., 2012; Song et al., 2012). Continuous measurement will allow researchers – in collaboration with therapists – to make very fine-grained observations of couples’ co-thermoregulatory responses. Pairing these co-thermoregulatory mechanisms to relationship outcomes (e.g., marital dissolution, relationship satisfaction) will help us classify clients’ thermoregulatory responses as responsive or unresponsive.

Having sensor technology thus resolves a number of problems that researchers in psychology typically encounter, like lack of measurements. While psychologists typically focus on confirmatory studies, little sensible hypotheses can be formulated regarding the topic of co-thermoregulation. To create social thermoregulatory interventions, we thus advocate focusing on *descriptions* of relationships first, *without* predefined models. The idea is to measure couples in their daily lives; doing so across different situations across different relationships then allows for specifying which co-thermoregulatory patterns define high quality relationships. This means that (1) we quickly come to understand *whether* co-thermoregulation predicts relationship success, (2) which types of emotions rely on co-thermoregulation (and in which types of situations) and (3) which are the most ideal patterns to oscillate, for which types of individuals. Such approaches will thus allow us to quickly gain ground, create more accurate models, and from there design (confirmatory) randomized control trials. Furthermore, when mechanisms are understood based on exploratory research and Randomized Clinical Trials (RCTs) first and protocols developed second, such an approach will allow therapists to become more client-centered, as the increased amount of measurements will afford a greater focus to study clients at the intra-individual level (Whitsett and Shoda, 2014; LeBel et al., 2016).

### Measurement

**FIGURE 1:**
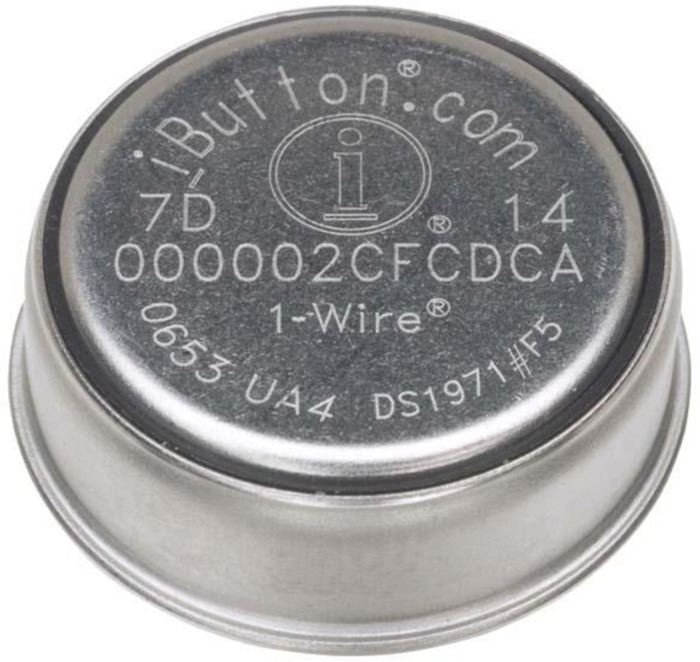
**Maxim’s “Thermochron iButton DS1291H.**”

In our own research, we have focused on using devices that are as non-intrusive as possible. Thermoregulation researchers consider the gold standard in measuring peripheral temperature Maxim’s “Thermochron iButton DS1291H,” which has a mean accuracy of -0.09°C and a precision of 0.05°C (Van Marken Lichtenbelt et al., 2006; Pouw et al., 2012; see also **Figure 1**). The advantage of the iButton is that it is wireless and can be easily attached to one’s body. The downside of the iButton is that it is impractical in daily life as it is attached to the skin at the finger or arm.

**FIGURE 2:**
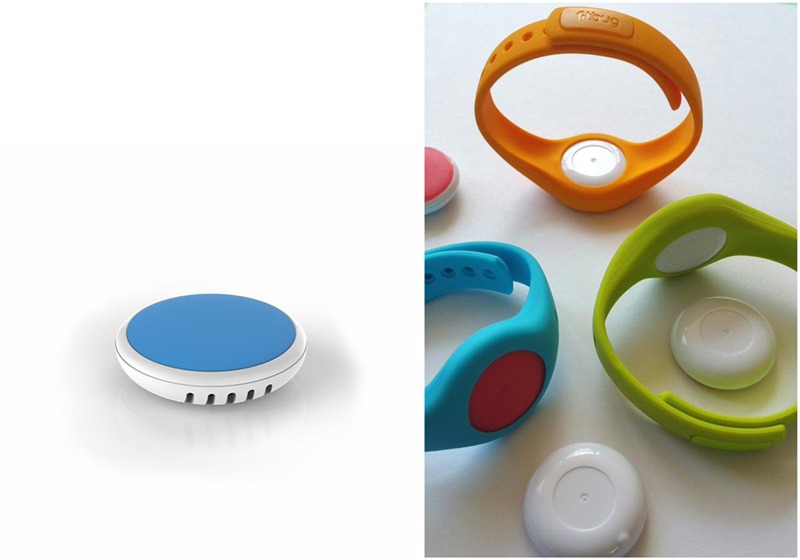
**BlueMaestro TempoDisc (left panel) and BlueMaestro TempoDisc in a Fitbug wristband (right panel) as used in our research**.

In our more recent research, we therefore chose to move to a different sensor, the BlueMaestro Tempodisc (see **Figure 2**), which also has a precision of 0.05°C (but which is still in need of independent verification). The advantage of the BlueMaestro Tempodisc is that it can be inserted in a FitBug wristband and can be easily worn in daily life. Additionally, the BlueMaestro Tempodisc can connect to a smartphone via Bluetooth Low Energy and store and communicate the temperature changes in the wrist via our “Temperature Detection App” to our central server (for our present version, see IJzerman et al., 2017a). The sensors are affordable and our software open source.

To apply these sensors for measurement in research during EFT, couples can wear bracelets with a temperature sensor that connects to their own mobile phones via Bluetooth Low Energy. In order to be able to classify the thermoregulatory dynamics, therapists and researchers can then assess their clients’ co-thermoregulation in their day-to-day life and connect these to clients’ discrete emotional states via smartphone apps (e.g., through the SurveyMonkey or MoodiMoodi, app, etc.), and proximity to one another via Bluetooth connection or GPS.

Beside the BlueMaestro TempoDisc, a multitude of sensors are becoming available to measure peripheral temperature, such as a *thermodo* (a tiny thermometer one can plug into one’s smartphone), and several skin thermometer gadgets (Coxworth, 2013; see **Table 1** for a list of several possible technological sensors to be used in research and therapy). The challenges to create STT are obvious, but become resolvable: beyond needing to interpret just how thermoregulation relates to discrete emotional states, it is unclear *how quickly* a thermoregulatory response to the other is most efficacious. Furthermore, *how strongly* can and should a partner respond to the other’s distress? It is clear that the understanding of many of the mechanisms we outline here are in their beginning stages. But the exploratory approaches we pointed to are an important first step to be able to create accurate descriptions of what are high quality relationships. *Data-driven approach* will help us classify which thermoregulatory responses relate to “healthy” relationships versus relationships for those in need of therapy.

**Table 1:** Specifications of possible sensor devices to be used in co-thermoregulation research and therapy.

	Range (° C)	Accuracy (° C)	Resolution (°C)	Data Transfer Method	Independently Verified?	Data saved on own server only? If no, where?
Tempodisc	-25 to 75	±0.5	Unknown	Bluetooth Low Energy to smartphone app	No	No, BlueMaestro server
iButton	-20 to 85	±0.5	0.625	BlueDot receptor attached to PC	Yes	Yes
Tokyo University Sensors	25 to 50	±0.1	Unknown	Unknown	No	Unknown
MIT Band-Aid	Unknown	Unknown	Unknown	Unknown	No	Unknown
YSI 400	0 to 60	±0.1	Unknown	Unknown	Yes	Yes
Thermocouple Type T	-270 to 400	±1	Unknown	Wired	Yes	Yes
Thermistor	-100 to 300	±0.1	Unknown	Wired	Yes	Yes
Cyberglove II	10 to 45	3	<1	Wireless USB plug	No	Yes

For this, we favor prediction over explanation: by deriving predictions from data, we can thereafter start formulating theories on how to manipulate temperature and how to craft interventions. One powerful exploratory approach that allows for classification of co-regulatory systems and making predictions from data is *supervised machine learning* (Breiman, 2001; IJzerman et al., 2016; Yarkoni and Westfall, 2016). Machine learning relies on explorative algorithms to *learn* from data, deriving complex patterns as accurately and reliably as possible. Machine learning helps to deeper understand data and reduces, for example, problems of *under-* or *overfitting*, or the problem to apply models that are overly simplistic or complex and also prevents us from applying linearity where none exists (Boulesteix et al., 2012). Supervised machine learning can thus help us generate patterns where we have no reasonable predictions *a priori*. Such exploratory approaches thus hold great promise *specifically* for real world problems such as how to integrate STT into EFT. Thus, instead of having fixed hypotheses, patterns not defined *a priori* can be detected and hypotheses derived thereafter.

Supervised machine learning thus help *classify* which co-thermoregulatory patterns relate to successful relationship outcomes, and will help define what is responsive and what is unresponsive co-thermoregulation. Applying this approach to psychological science, IJzerman et al. (2017b) classified complex social integration as one of the most important predictors of core body temperature. Using a similar approach, researchers and therapists can easily identify whether responsive co-thermoregulation is one of the most important predictors of relationship quality (or not), and which types of oscillation patterns are ideal for high quality relationships. We suggest supervised over unsupervised machine learning, as the proposed research provides a so-called “supervisory signal” (e.g., whether people stay together or what they perceive the quality of their relationship to be).

Prior to intervention, several of such studies are needed to understand exactly how (and whether) communal relationships are facilitated through co-thermoregulation and how (and whether) interventions should be crafted to trigger *responsive co-thermoregulation* in couples that suffer from *unresponsive co-thermoregulation*. Research needs to be conducted to understand *how* therapists can intervene to train couples to show greater responsive co-thermoregulation in case the therapist decides he or she should help the couple. But whether this is efficacious, whether this is helpful in the relationship, and whether this is helpful to the individual needs to be researched carefully through collaborations between researchers and therapists. Finally, measuring subjective experience and expressive behavior are of course crucial to fully appreciate the *relative* contribution of STT in comparison to other therapies (see, for example, the development of an algorithm for our baby app that can detect and record the crying of infants; Lavner et al., 2016).

### Intervention

Once exploratory approaches are finished, protocols for therapy can be tested in to-be designed randomized controlled trials (RCTs). Such RCTs can lead to interventions, through tactile technological devices, and we believe these could be available in the near future. One of the most promising devices for intervention is the “Wristify,” a wristband that can manipulate peripheral temperature. In our own research, we currently use a design inspired by the Wristify, with actuators integrated into a bracelet that holds a Peltier element (controlled through an Arduino Uno with a Velleman VMA23 Motor Shield) that can apply alternating pulses of hot or cold to the skin with a range of 0.4°C per second. The pulse provides a strong subjective experience of feeling warmed or cooled. Because the wristband can be worn and controlled through Bluetooth Low Energy, with sufficient understanding of co-regulatory dynamics, apps can be designed to apply interventions in daily life. These interventions can be tailor made by the therapist for the client and controlled and monitored from a distance. We suspect interventions will be focused on enhancing a more permanent perception of the relationships’ predictability (i.e., the communality) through *associative learning* (Beckes et al., 2013).^9^ The Wristify is but one of the technologies; we have summarized some relevant technologies for intervention in **Table 2**. Besides wristbands to warm up or cool down, several companies have been experimenting with game controllers using temperature feedback (Dillow, 2010; Fincher, 2012).

**Table 2:** Specifications of possible actuator devices to be used in co-thermoregulation research and therapy.

	Speed / Efficiency (°C)	Method of Manipulation	Independently Verified?
Wristify	0.4 per second	(warm/cool) pulses	Yes
MIT Band-Aid	Unknown	Unknown	No
Thermosuit	-3 core temperature in 30 min	Full-body suit with waterfilled tubes	Yes
Sensor and actuator by University of Illinois	Unknown	Warming skin on top of vein	No
Electronic Skin	Unknown	Microheater on skin	Yes
Climaware Wrist Wrap	8 to 43 in a few seconds	Cools/heats wrist	No
Sony – Temperature feedback motion controller	Unknown	Cold/hot grip in hand, also fan could expel cold/hot air	No
Powerclaw haptic gloves	Unknown	Gloves with actuators	No

Thus, through actuators built into a wearable device, unresponsive co-thermoregulation could be manipulated to be responsive so as to support couples that have relationship problems. One option might be to give warm (or cold) pulses to one’s skin, like the wrist, with a tactile device when one’s partner is sad (or otherwise shows a peripheral temperature drop) to upregulate one’s temperature that we suspect will help regulate one’s partner.^10^ We again stress that the exact mechanisms are still unclear and that STT should not be integrated into relationship therapy until a number of exploratory and confirmatory studies have been conducted.

## The Risks of Relying on Big Data and Further Ethical Concerns

With such potential for rapid change and advances, we also see considerable risks. First, careful (theoretical) interpretation of data is a dire necessity and not *just* relying on automatic classification through machine learning. Without interpretation, automated processes may become unfair to one of the relationship partners, or evidence may be misguided based on pre-existing biases in past research. For example, suppose stigmatized couples or couples from lower socio-economic status do not benefit from co-regulation as others, researchers may infer that they are unable to co-regulate. However, it could instead be that the inability to co-regulate or to benefit from it is caused by perceived threat in the environment, rather than an inherent inability. An intimate collaboration between therapists and researchers to interpret complex data through a theoretical lens will be required to prevent such mishaps.

Furthermore, even though therapy may become cheaper and the threshold to seek therapy lower, future clients may fear intrusions of their privacy, with manipulations of their personal life in ways they do not desire – for good reasons. Leaked records of *Big Data* now total over 30,000 records (World’s Biggest Data Breaches, 2016), while, amongst others, pharmaceutical companies (Hardekopf, 2015), real estate companies (Shamah, 2015; Ward, 2015), web shops (Marr, 2015), and Google (Marr, 2015; Van Rijmenam, n.d.) make use of *Big Data* for commercial interest in ways not necessarily for the interest of the consumer. Furthermore, rumors of the 2016 American election being manipulated by Russian hackers or companies like Twitter being brought offline through an attack on everyday wireless devices are real and legitimate concerns. One could only imagine the nightmares associated with an industry focused on manipulating and controlling one’s social network. Thus, forethought for how to handle data from therapy is required and solid privacy and security protocols need to be created (Liu and Kuhn, 2010).

As a first step, the European Union now has adopted a code of conduct on privacy for mobile health applications, which specifies general guidelines for data storage (e.g., not store exact age of birth), including the “right to identity” and specifies what to do in case of data breaches (European Commission, 2017). How to prevent data breaches is still in its infancy, and the discussion on data breaches should become an important part of being able to use *Big Data* for STT.

Beyond legitimate concerns about novel technologies and questions of privacy, people may also be wary to start a therapy using novel technological devices, as fear and distrust tend to emerge at the introduction of novel technologies (q.v., Marshall, 2014; Wilson, n.d.). To avoid this, therapists need to foresee and be responsive to users’ fears and developers need to design the technologies (a) to anticipate and consider the expectations, fears, and values of therapists and clients (b) to most naturally integrate them into clients’ daily lives (q.v., Bartneck et al., 2007; Mori et al., 2012; Canepari et al., 2015).

To help integrate such technologies, it will be helpful to create educational material such as introductory videos demonstrating how and why the devices are used, while test booths can be created for potential new participants to test devices and instruct clients before starting the study or therapy. This allows the user to maintain control over the intervention and feel empowered to stop its usage when desired. Further, manipulations in day-to-day life of one’s body may feel intrusive and may let the client wonder whether the relationship *is* still authentic, and importantly: *perceived* as authentic. Another relevant question for the balancing of benefits, risks, and costs of an intervention is whether couples can be aided by technological devices to, from there on, sustain the responsive co-thermoregulation on their own, or whether they will need the technology as a constant aiding device in times of need?

### Responsible Implementation of Technology in Therapy

A key question that emerges is how to responsibly introduce such technology in the therapeutic relationship. Besides the effect monitoring may have on the person’s behavior and feelings, implementation may also have effects on relationships between client(s) and therapist. Importantly, the therapist using this intervention must be sensitive in understanding the potential effects of the device on the single client, the couple, as well as on the relationship between the client/couple and the therapist. Does the technology support a trustful therapeutic relation or hinder it? How can the therapist understand whether the intervention is helpful versus harmful? Here, concerns about authenticity, naturalness, and autonomy may continue to arise. These need to be addressed by the therapist before using the devices, and they should be considered in the design phase of the devices already. And there are practical challenges with the usage in continuously monitoring in real life: what if, for example, the therapist’s responsibility when he or she suspects or even notices through suspicious patterns from the fine-grained data that her client is cheating on his or her significant other?

## Conclusion

There are still a number of questions that need to be answered before one can intervene through STT. We nevertheless aimed to provide a convincing case for its need. We have first shown considerable evidence that thermoregulation is still key to people’s modern social lives and we have discussed existing evidence on co-regulation. From there, we integrated the literatures, arguing that responsive *co-thermoregulation* is a crucial feature of a healthy emotional social life. We have discussed the limitations of what we know and don’t know, and the path to crafting a responsible STT. Clearly, research in the area of co-thermoregulation is still in its beginning stages. However, with the current theoretical knowledge and advancements within technology and statistical analyses, like actuator and sensor technologies and supervised machine learning, new research can be conducted with greater reliability, accuracy, and replicability. We suspect that STT can become an important part of how we improve our relationships, and that STT will become integrated into EFT for maximal effectiveness.

Technologies have become available and researchers sensitively need to help channel the implementation of these technologies, discussing the benefits and perils to allow responsible innovation. First exploratory studies – combining teams of researchers from different disciplines with therapists – need to be conducted to assess how, in whom, when, and where co-thermoregulation works. Based on this, RCTs should be designed using haptic technologies (see **Table 2**) to see whether and how, when, and with whom interventions are possible and beneficial when deemed necessary. This challenge is worthwhile because loving and warm relationships are not only pleasant, but will lead to a longer, happier, and healthier life.

## Author Contributions

HIJ and EH: wrote the first drafts of the paper. SN and TP: provided several critical revisions of the paper and helped writing the paper, and HIJ: finished the final version of the paper. HIJ: mostly wrote the revisions after the first submission, where TP and SN: provided several critical revisions.

## Conflict of Interest Statement

The authors declare that the research was conducted in the absence of any commercial or financial relationships that could be construed as a potential conflict of interest.

## Footnotes

1 The field of social thermoregulation in humans is its infancy. Nevertheless, a considerable amount of evidence has been gathered on the relationship between temperature regulation and social behavior. This does not mean that this field is without its criticism (and rightfully so). Given the discussion on power in psychological science, it may then also not come as a surprise that also in the field of thermoregulation some effects failed to replicate (Vess, 2012; LeBel and Campbell, 2013). Yet, other effects did replicate (IJzerman and Semin, 2009; Schilder et al., 2014; Inagaki et al., 2015; Ebersole et al., 2016; IJzerman et al., 2016). Further, original studies with larger *N*s do exist, with some studies with participant samples between 100 and 500 (e.g., IJzerman et al., 2015b; Van Acker et al., 2016), and some outliers even with samples around 30,000 (Hong and Sun, 2012) and above 6 million (Zwebner et al., 2013). We think that the criticisms should likely be directed at better specifying the models relevant for social thermoregulation theory, for which we see this paper as an important step in the right direction.

2 Note that the more dynamic view of social thermoregulation diverges substantially from what one may understand as *conceptual embodiment*, a view advocated by for example Lakoff and Johnson (1999). They propose that warmth becomes paired with affection at an early age, and that such peripherally related constructs form a mental representation of relationships. Our view instead relies on more dynamic, and innate, co-regulation systems for which the infant searches from birth on, and that it *may* form an internal mental representation of its social network, scaffolded onto such early innate predispositions to search for warmth (cf. Bowlby, 1969; Mandler, 1992).

3 We would like to note that when we discuss peripheral temperatures here, we mostly talk about the extremities. Little is known about temperature changes throughout the body in response to social situations, but temperature changes in the extremities are for example likely to differ from temperature changes in the face. Indeed, social exclusion has been found to lead to decreases in the extremities (IJzerman et al., 2012) but increases in the face (Paolini et al., 2016).

4 Furthermore, the evidence on physiological patterns converging with social thermoregulation (like oxytocin and serotonin) converge with these ideas on co-thermoregulation (e.g., Beckes et al., 2015; Raison et al., 2015).

5 We have not discussed the differences between core and peripheral temperature. Core temperatures are relatively stable, although they are influenced by time of day, distance from the equator, sex, and the quality of one’s social network. Peripheral temperature is much more prone to change throughout the day. For example, peripheral temperatures drop when environmental temperatures drop and even drop about 0.7° after being socially excluded. This is so because the periphery serves as a defense mechanism from changes to the core.

6 We stress that the relationship between social thermoregulation and health to date has only been found in correlational studies, and no prospective studies have been conducted.

7 We have not even scratched the surface of the interrelationship between peripheral temperature changes and facial expression of emotions. We think it is likely that peripheral temperature changes are connected to facial expressions and other manifestations of emotions, which are thus dynamically connected to co-regulate emotions. Furthermore, we also have not even considered the link between individual differences in empathy or perspective taking. We suspect such relationships to exist, but how these links should be understood is beyond the scope of this article.

8 This is likely true because the regulation of body temperature can be understood asymmetrically: decreases in temperature are regulated socially, while this is not true of increases in temperature. This is highly contingent on the asymmetry of thermoregulatory systems: increases in core temperature are immediately dangerous for body and brain, whereas this is not true for decrease in temperature (thermosensitive neurons that detect increases in temperature are also more prominent in the hypothalamus, while thermoreceptors detecting coldness are more prominent in the skin; Hensel, 1974; Guyton, 1991). The idea of this asymmetry is also confirmed in the experimental literature: priming of trustworthiness (versus agency) in relationships leads to temperature increases under colder ambient conditions, but not under warmer ambient conditions (Ebersole et al., 2016; IJzerman et al., 2016). In addition, co-thermoregulation is certainly not the *only* aspect of co-regulation. Specifically, research across (human and non-human) animals also shows the importance of risk distribution (Ebensperger, 2001; Coan et al., 2006).

9 Although some have suggested that full-body warmth treatments can be effective against psychopathologies (Janssen et al., 2016), others have voiced their criticisms (Fried, 2016), and rightfully so. Social Thermoregulation Therapy is not *just* about warmth, but by associating warmth with predictability in the right social situations.

10 We again would like to stress that it will not *just* be thermoregulation that will help regulate the partner, but we postulate that thermoregulation is causally linked to other emotional states that allow for more direct regulation mechanisms.
